# K-Nearest Neighbor and Random Forest-Based Prediction of Putative Tyrosinase Inhibitory Peptides of Abalone *Haliotis diversicolor*

**DOI:** 10.3390/molecules26123671

**Published:** 2021-06-16

**Authors:** Sasikarn Kongsompong, Teerasak E-kobon, Pramote Chumnanpuen

**Affiliations:** 1 Interdisciplinary Graduate Program in Bioscience, Faculty of Science, Kasetsart University, Bangkok 10900, Thailand; sasikarn.k@ku.th; 2 Department of Genetics, Faculty of Science, Kasetsart University, Bangkok 10900, Thailand; 3 Omics Center for Agriculture, Bioresources, Food and Health, Kasetsart University (OmiKU), Bangkok 10900, Thailand; 4 Department of Zoology, Faculty of Science, Kasetsart University, Bangkok 10900, Thailand

**Keywords:** anti-tyrosinase peptides, bioinformatics, machine learning, random forest, k-nearest neighbor, abalone

## Abstract

Skin pigment disorders are common cosmetic and medical problems. Many known compounds inhibit the key melanin-producing enzyme, tyrosinase, but their use is limited due to side effects. Natural-derived peptides also display tyrosinase inhibition. Abalone is a good source of peptides, and the abalone proteins have been used widely in pharmaceutical and cosmetic products, but not for melanin inhibition. This study aimed to predict putative tyrosinase inhibitory peptides (TIPs) from abalone, *Haliotis diversicolor*, using k-nearest neighbor (kNN) and random forest (RF) algorithms. The kNN and RF predictors were trained and tested against 133 peptides with known anti-tyrosinase properties with 97% and 99% accuracy. The kNN predictor suggested 1075 putative TIPs and six TIPs from the RF predictor. Two helical peptides were predicted by both methods and showed possible interaction with the predicted structure of mushroom tyrosinase, similar to those of the known TIPs. These two peptides had arginine and aromatic amino acids, which were common to the known TIPs, suggesting non-competitive inhibition on the tyrosinase. Therefore, the first version of the TIP predictors could suggest a reasonable number of the TIP candidates for further experiments. More experimental data will be important for improving the performance of these predictors, and they can be extended to discover more TIPs from other organisms. The confirmation of TIPs in abalone will be a new commercial opportunity for abalone farmers and industry.

## 1. Introduction

Melanin is the primary determinant of skin, hair, and eye color, and helps protect against UV radiation [1]. Two types of melanin are produced in mammals; eumelanin (brownish black) and pheomelanin (reddish yellow) [2]. The production of melanin is catalyzed by a key enzyme, tyrosinase, known to be associated with pigmentation disorders [3]. Accumulation of this enzyme results in an excess amount of melanin and can cause freckles, lentigo, age spots, and skin cancer [4].

Many tyrosinase inhibitors have been used as skin whitening agents in the cosmetic industry, e.g., hydroquinone, kojic acid (KA), or arbutin. Their use remains limited due to the side effects of skin irritation, low stability in oxygen and storage, cytotoxicity, and insufficient skin penetration ability [5,6]. Investigation of new natural compounds has provided alternative opportunity for the industry. Several non-cytotoxic natural tyrosinase inhibitory peptides have been identified in the last 10 years, including proteins and peptides from milk, wheat, honey, and silk [6,7,8,9,10,11,12]. A diverse array of peptides have tyrosinase inhibitory functions such as cyclic peptides [13,14], *N*-acetyl-pentapeptides [15], mimosine-tetrapeptides [16], kojic acid-peptides [5,17], and dipeptides [10]. These tyrosinase inhibitory peptides (TIPs) are hypothesized to reduce the melanogenesis process by inhibiting tyrosinase activity. Free amino acids, like cysteine, are also known to be one of the best tyrosinase inhibitors [18]. Strong tyrosinase binding peptides normally contain at least one arginine or phenylalanine with valine, alanine, and/or leucine, as well as those with hydrophobic properties [7].

Abalone is a highly valued nutritious food and luxurious cuisine [19,20,21] and has valuable bioactive molecules with anti-thrombotic, anticoagulant, anti-inflammatory, antioxidant, and anticancer properties [22]. However, the anti-tyrosinase property of abalone peptides has never been reported. Experimental identification of TIPs is costly and time intensive. Computational methods thus provide a preliminary solution to this hypothesis. Nithitanakool et al. [23] used molecular docking to examine the effect of compounds from Thai mango seed kernel extract on tyrosinase binding and predict anti-tyrosinase ability. Many studies have used machine learning (ML) methods to create prediction tools for various peptide properties such as MLACP for anticancer [24], AIPpred for anti-inflammatory [25], anti-biofilm [26], AmPEP for antimicrobial [27], and cell penetrating [28]. These tools were developed by extracting properties including amino acid composition (AAC), dipeptide composition (DPC), and physiochemical composition (PCP), from the amino acid sequences and used them as input features to train random forest (RF), k-nearest neighbors (KNN), and support vector machine (SVM) algorithms. Currently there are no anti-tyrosinase prediction tools. This study aimed to develop the TIP prediction tool based on the integration of k-nearest neighbor (kNN) and random forest (RF), and to predict the TIPs from abalone peptides of *Haliotis diversicolor*. Our previous study identified thousands of the abalone peptides from proteomic experiments. Discovering abalone putative anti-melanogenesis peptides and further characterizing them will be interesting for application in the pharmaceutical and nutraceutical industries.

## 2. Results

**Figure 1:**
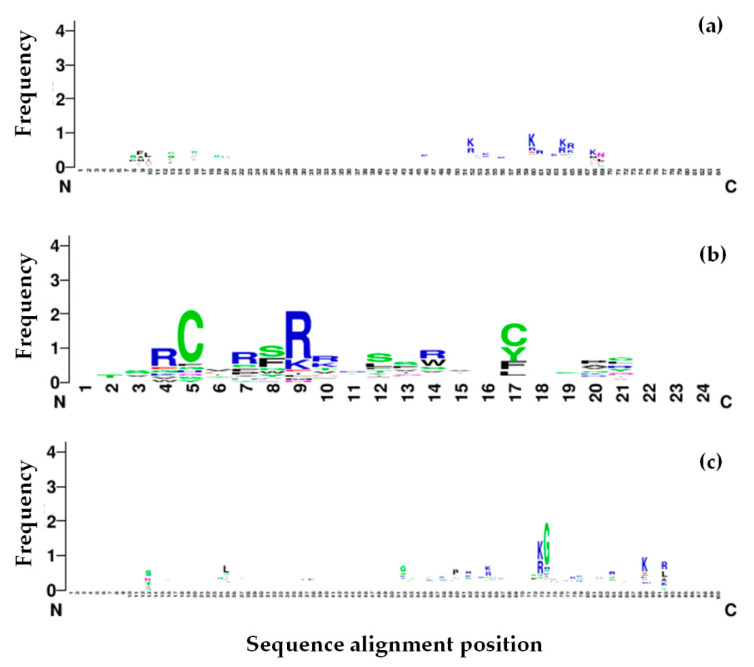
Multiple sequence alignment of known tyrosinase inhibitory peptides (TIPs), predicted TIPs and non-TIPs represent by the logo character plot. (**a**) predicted TIPs; (**b**) known TIPs; and (**c**) non-TIPs. Height of the amino acid characters showed the frequency that they appeared in the peptide sequences at a particular position.

One-hundred and thirty-three known anti-tyrosinase peptides were obtained from literature mining of 13 published research articles. These peptides were successfully used to develop kNN and RF-based TIP predictors. Performance measurement of these two predictors on the test dataset showed high accuracy, sensitivity, specificity, precision, recall, and receiver operating characteristic curve (ROC) scores (Table 1). However, the area under the curve (AUC) scores were 0.08 for the kNN model and 0.02 for the RF model. The kNN classifiers predicted 1075 TIPs and the RF classifiers predicted six TIPs from 8330 abalone peptides (Table S1). Fifty-eight peptides (5.4%) had a kNN predictive probability score of 1.0 and 758 peptides (70.5%) had scores more than 0.9. Two of six peptides identified from the RF classifier were also predicted by the kNN classifier with probability scores of 0.77 and 0.5. The first peptide (TIP1) had nine amino acids with double serine residues, two aromatic residues (tryptophan and tyrosine), one negatively charged aspartic acid, and one positively charged arginine (TASSDAWYR). The second peptide (TIP2) was 13-amino acids long with double phenylalanine residues, one negatively charged aspartic acid, and one positively charged arginine (SAPFMPDAFFRNV). Peptide sequence alignment of all predicted TIPS showed frequent patterns of positively charged residues (arginine and lysine) similar to those of the known TIPs, which showed frequent occurrence of arginine, lysine, cysteine, and serine (Figure 1). On the other hand, the non-TIPs had a frequent pattern of glycine in addition to the arginine and lysine appearance.

**Figure 2:**
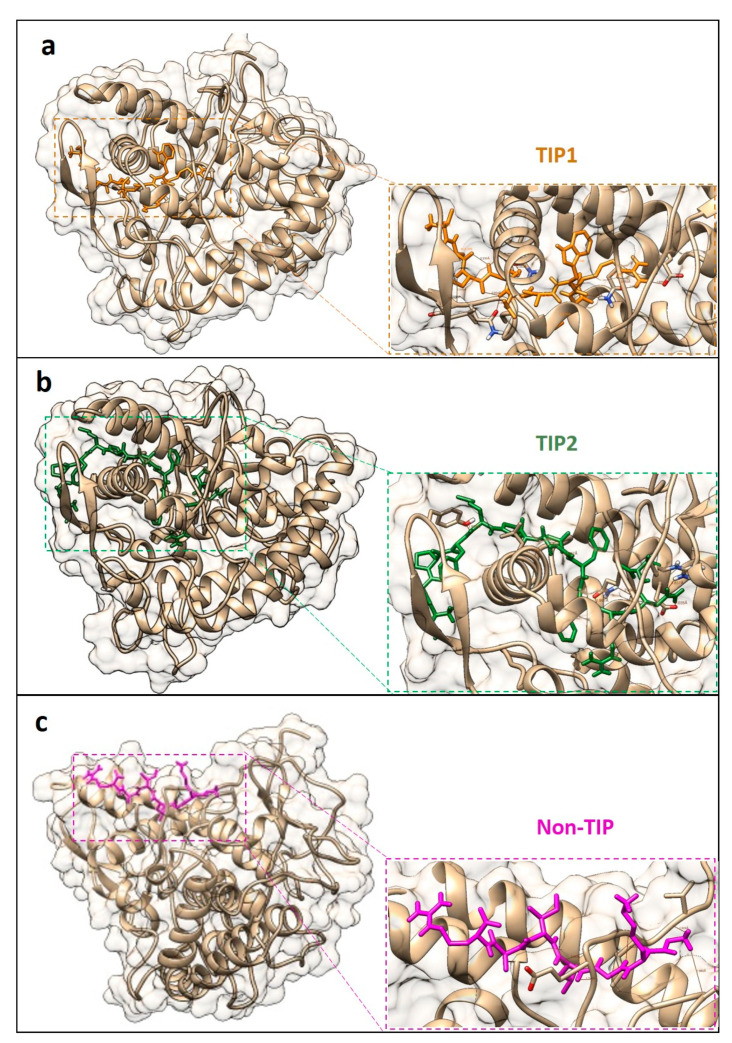
Molecular docking of two tyrosinase inhibitory peptides (TIP1 (**a**) and TIP2 (**b**)) and non-tyrosinase inhibitory peptide (non-TIP (**c**)) to the crystal structure of mushroom tyrosinase (PDB ID: 2Y9X). Structure of the mushroom tyrosinase is shaded in gray and the peptide sequences are colored as labeled above. The hydrogen bonds are shown as black lines.

**Figure 3:**
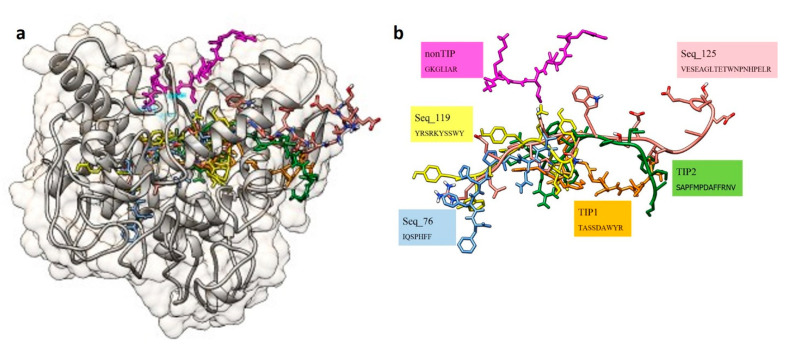
Comparative molecular docking of three known tyrosinase inhibitory peptides (Seq_76, Seq_119, and Seq_125) with those of the non-tyrosinase inhibitory peptide (non-TIP) and the putative tyrosinase inhibitory peptides (TIP1 and TIP2) on the crystal structure of mushroom tyrosinase (PDB ID: 2Y9X) shown with (**a**) and without (**b**) the enzyme structure. Structure of the mushroom tyrosinase is shaded in gray and the peptides are labeled with different colors.

Predicted structure of TIP1 and TIP2 showed alpha helical conformation. Molecular docking of these two TIPs to the predicted structure of mushroom tyrosinase demonstrated that TIP1 and TIP2 were similarly localized closer to the active site of the mushroom tyrosinase structure (as referenced by the position of the small ligand of the inhibitor tropolone in the structure) compared to those of the non-TIP, GKGLIAR (Figure 2). TIP1 could interact with eight residues near the active site of the mushroom tyrosinase by hydrogen bonding, while TIP2 interacted with six residues in the site (Figure 2 and Table 2). The non-TIP only interacted with six residues further out of the active site area (Figure 3). When compared with the interaction of the known TIPs (Seq_76, Seq_119, and Seq_125), the TIP1 and TIP2 showed binding positions in a similar manner to the known TIPs, and the non-TIP was clearly shown to be further away (Figure 3).

## 3. Discussion

Bioinformatics prediction of the tyrosinase inhibitory peptides is challenging due to lack of the TIP predictors available. This study has gathered the TIP information and found frequent amino acid patterns of these peptides consistent with those described by Schurink et al. [7]. This study observed that peptides containing at least one arginine, lysine, and phenylalanine would favor strong binding to the tyrosinase because of their charge properties, enabling the peptide–enzyme interaction (Figure 1). The finding could potentially support the predictive effort of the kNN and RF predictors, which attempted the preliminary prediction of the TIPs from limited amount of known data. The kNN algorithm is one of the simplest methods to classify the TIPs and non-TIPs by k nearest datapoints, in this case, the physicochemical properties and amino acid patterns. The peptides with similar feature patterns to those of the known TIPs would be expected to be closer than the distinct ones. For the RF predictors, multiple decision trees were generated from our features. Some features could contribute to building a set of decision trees specific for classifying TIPs from non-TIPs. Combination of one simple and another complex machine learning algorithm allowed us to gather possible TIPs at first by the kNN classifier and then finely narrowed with the RF. A thousand of the kNN-predicted TIPs were reduced to a manageable number of six peptides by the RF classifier. The classification performance of these two algorithms were also recommended to use with various data types over Naïve Bayes algorithm by Singh et al. [29]. Despite the low AUC values of these two predictors, they might have been disregarded as giving more chance to the false positive predicted TIPs and deriving from an unbalanced dataset and effect of the oversampling method, leading to overfitting of one classifier to the data. However, the classifiers of this study had only the TIP and non-TIP group, and the performance for separating the TIPs was reasonably high based on other parameters, as shown in Table 1. Having bias towards one or another group would remain beneficial to the prediction because the authors have to interpret the predicted results by comparing the peptides with known properties from different experimental results. Fortunately, the predicted TIPs in this study had shared some sequence patterns with the known TIPs, although some might be lost as false negatives. These kNN and RF predictors proposed a few TIP candidates from nearly 8500 abalone peptides. These candidates will be easily examined by peptide synthesis and in vitro experimental treatment with tyrosinase or tyrosinase-producing cell lines. Further experimental results will assist the improvement of the TIP predictors. Successful detection of putative TIPs from the abalone peptides has raised a possible hypothesis on how the peptides performed the inhibitory function. Several organic compounds have been shown to be tyrosinase inactivators and inhibitors, scavengers of intermediate compounds, and denaturants [30]. From our molecular docking result in Figure 2, TIP1 and TIP2 were likely to be either competitive or non-competitive inhibitors of the tyrosinase, which bound to the external helixes and perhaps affected conformational change of the enzyme during the reaction, resulting in a reduction in melanin production. This study also compared the predicted TIPs and non-TIP to the interaction of the known TIPs (seq_76 (IC50 of 1.7 mM for monophenolase and 4.0 mM for the diphenolase activity) [12], seq_119 (IC50 of 40 µM) [8], and seq_125 (IC50 of 0.1 mM) [6]. Docking positions closer to the active site of the mushroom tyrosinase structure were very similar between the known TIPs and our predicted TIP1 and TIP2, compared to the non-TIP. The similar binding area of TIP1 and TIP2 to those of the three known TIPs could be further in silico evidence suggesting possible tyrosinase inhibition of our predicted peptides. Shen et al. [31] examined the inhibitory reaction of the similar peptide ECGYF (with two aromatic residues of tyrosine and phenylalanine) on tyrosinase activity, and their CD spectrometric analysis suggested that the peptide could bind to the non-active site of tyrosinase and alter the enzyme conformation, hence interfering with melanin synthesis. Therefore, the first version of the TIP predictors could suggest a reasonable number of TIP candidates for further experiments. More experimental data will be important for improving the performance of these predictors, and they can be extended to discover more TIPs from other organisms. The confirmation of TIPs in abalone will be a new commercial opportunity for abalone farmers and industry.

## 4. Materials and Methods

**Figure 4:**
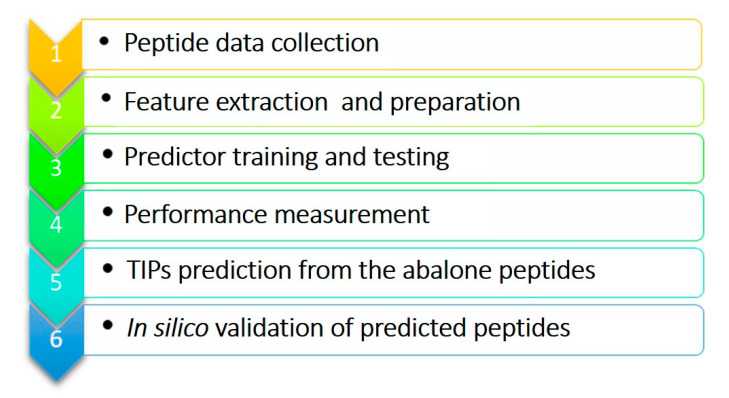
Workflow for bioinformatics prediction and in silico validation of the TIPs from abalone peptides.

The overall workflow of this study is summarized in Figure 4. Peptides with known tyrosinase inhibitory properties were collected from previously published research [6,7,8,9,10,11,12,29,32] and the peptide sequences were prepared before using the predictor development. The peptide sequences were used as input for the in-house written R scripts to calculate amino acid (20 features) and di-amino acid composition (20 × 20 features), hydrophobicity, peptide length and mass, and numbers of positive charge and negative charge residues, and convert to a numeric matrix of 425 features.

The amino acid composition (AAC) of amino acid *i* was calculated in the equation below [26,33].

(1) $$AAC\left(i\right)=\frac{\;Amino\;acid\;frequency\left(i\right)}{Peptide\;length}$$

Di-amino acid composition (DAA) represented the total number of dipeptides divided by 400 possible dipeptides in the given peptide sequence. The DAA of dipeptide *i* was calculated using the following equation [25,34,35].

(2) $$DAA\left(i\right)=\frac{Total\;number\;of\;dipeptdes\;\left(i\right)}{Total\;number\;of\;all\;possible\;dipeptides}$$

Physicochemical properties of the peptides were calculated from the percentage composition of hydrophobic (C, F, I, L, M, V, W), positively charged (K, R, H), and negatively charged (D, E) amino acid residues [24]. A new column was added to label the known TIPs as antimelanogenesis and non-TIPs as non-antimelanogenesis.

The k-nearest neighbor and random forest-based predictors were created by using the R scripts. The kNN performed a pairwise computation of a certain distance or similarity measure (k-value) for each unknown sample on every training sample [36]. This method classified the samples into groups by choosing the nearest group to the unknown samples based on the k-value [37]. The RF algorithm is suited for large datasets and has multi-model classification [34,35]. It consists of hundreds or thousands of decision trees that are called forests. Each forest randomly selects the feature at each node to determine the split and choose one or two features frequently given near the optimum results [25,38].

As the dataset was unbalanced, the oversampling method was used to balance the data with the ovun.sample() function of the ROSE package. The TIP/non-TIP dataset was split into 75% training and 25% test sets using the createDataPartition() function of the caret package. The training dataset was given to the knn3() function of the caret package with the optimized k value, k = 2 (from the optimization against the test dataset between k = 2 and k = 10), and randomForest() functions of the randomForest packages using ntree = 1000. The created predictors were tested against the test dataset using the predict() function and setting the type argument to “prob” for recording the predictive probability. Performance of the prediction was measured by calculating accuracy, sensitivity, specificity, precision, recall, receiver operating characteristic curve (ROC), and area under the curve (AUC) using the confusionMatrix() function, and the twoClassSummary() and prSummary functions of the MLmetrics package. These scores were calculated by the following equations.

(3) $$Accuracy=\frac{TP+TN}{TP+TN+FP+FN}$$

(4) $$Sensitivity/recall=\frac{TP}{TP+FN}$$

(5) $$Specificity=\frac{TN}{FP+TN}$$

(6) $$Precision=\frac{TP}{TP+FP}$$

The ROC was a plot between false positive rate (FPR) as an X axis and true positive rate (TPR) as a Y axis. FPR and TPR were calculated by the following equations.

(7) $$FPR=\frac{TP}{TP+FN}$$

(8) $$TPR=\frac{FP}{FP+TN}$$

where *TP* = true positive, *TN* = true negative, *FP* = false positive, and *FN* = false negative. The AUC score was the measurement of the area underneath the ROC curve.

The TIP candidates were subjected to three-dimensional structure prediction using the PEP-FOLD program [39]. Peptide sequences of the known TIPs, predicted TIPs, and non-TIPs were multiply aligned using the ClustalW algorithm in the MEGA-X program version 10.2.2 [40]. The aligned sequences were visualized by plotting the logo graph using the WebLogo program version 2.8.2 [41]. Protein crystal structure of the mushroom tyrosinase (PDB ID: 2Y9X) was obtained from the PDB databank [42]. The predicted structures of known TIPs (Seq_76, Seq_119, and Seq_125), predicted TIPs and non-TIPs were docked to the mushroom tyrosinase enzyme using the GalaxyPepDock (http://galaxy.seoklab.org/pepdock/ (accessed on 12 May 2021)) and the observed interactions were compared [43]. The docking results of the best model and hydrogen bond finding were visualized by the UCSF Chimera program to ascertain the putative predicted TIPs [44].

## 5. Conclusions

In conclusion, this study proposed using the first version of the kNN and RF-based TIP predictors to obtain two TIP candidates from 8330 abalone peptides for further experiments. TIP1 and TIP2 shared similar in silico binding activities to the known TIPs. The predictors can be extended to discover more TIPs from other organisms. The experimental validation of the abalone TIPs will provide novel commercial opportunity for abalone farmers and industry.

## Figures and Tables

**Table 1:** Performance measurement of kNN and RF-based TIP predictors on the test dataset evaluated by the confusionMatrix() function of the caret R package.

Machine Learning Prediction Algorithms	Performance Measurement					
	Precision	Recall	Accuracy	Sensitivity	Specificity	ROC ^1^
kNN	0.89	1.00	0.97	1.00	0.96	1.00
RF	0.97	1.00	0.99	1.00	0.99	1.00

^1^ Receiver operating characteristic curve.

**Table 2:** List of hydrogen bonds observed from molecular docking of three peptides (TIP1, TIP2, and non-TIP) to the crystal structure of mushroom tyrosinase (PDB ID: 2Y9X).

Peptides	Peptide Residues	Tyrosinase Residues	Distance (Å)
TIP1	SER 3	GLU 160	1.981
	SER 5	ASN 173	2.402
	SER 4	GLN 43	2.053
	TRP 7	GLN 132	1.901
	ARG 9	GLN 132	2.023
	ARG 9	GLN 132	1.939
	ARG 9	GLU 97	1.898
TIP2	ASP 7	LEU 34	1.952
	ARG 11	GLN 132	1.991
	ASN 12	GLN 132	2.096
	ASN 12	ARG 19	1.857
	ASN 12	GLU 97	2.035
Non-TIP	GLY 1	ILE 12	1.832
	GLY 1	GLY 11	1.924
	GLY 1	THR 359	1.980
	LYS 2	PRO 13	1.903
	LEU 4	ILE 16	1.746

## Data Availability

Not applicable.

## Supplementary Materials

The following are available online. Table S1: Abalone predicted anti-TIPs by kNN and RF-based predictors.
