# Supplementary material for: K-Nearest Neighbor and Random Forest-Based Prediction of Putative Tyrosinase Inhibitory Peptides of Abalone Haliotis diversicolor
Source: Molecules. 2021 Jun 16;26(12):3671. doi: 10.3390/molecules26123671 (PMC8234169; doi:10.3390/molecules26123671)
Supplement: Supplementary file 1 [file molecules-26-03671-s001.zip › molecules-1243646-supplementary.pdf]

Table S1 Abalone predicted anti-TIPs by kNN and RF-based predictors

		Prediction probability	
No	Peptide sequence	kNN predictor	RF predictor
1	TASSDAWYR	0.97	0.71
2	SAPFMPDAFFRNV	0.79	0.54
3	SAPTFFR	0.00	0.63
4	NSSLRVQSR	0.00	0.60
5	SQSNSRSVSR	0.00	0.52
6	SALDSLL	0.00	0.51
7	NICECMK	1.00	0.39
8	TSQMSRSSSR	1.00	0.37
9	KKNYRVSEAYK	1.00	0.32
10	SASCSQSRSRSRER	1.00	0.10
11	ASRSGSSRRK	1.00	0.10
12	RGNEMCKK	1.00	0.08
13	GGEGGGKGRGGERGGEGR	1.00	0.07
14	QFVRNGFEHWGSDHQGVETTRRFLLEMLSFSHR	1.00	0.06
15	NPRYYGQGGEDEGSFR	1.00	0.06
16	VPTNSSNCNCRNPNTCEVEGNCKMK	1.00	0.06
17	SIPCALK	1.00	0.06
18	MGLAIQSLAELLIVR	1.00	0.06
19	ICAEKKVGSAICK	1.00	0.05
20	YFFIFYLCK	1.00	0.05
21	DKLLEFLPAMTRNLSFYDVAEITK	1.00	0.04
22	SRSSSRSSSGK	1.00	0.04
23	ELINEILSDTKFLAAIQEHNPEFFVVDNFMYIRNILV	1.00	0.03
24	LACKKIMEDR	1.00	0.03
25	SNSPTNMSK	1.00	0.03
26	SRSKSGSRSR	1.00	0.03
27	MGSRFFMSLLSMLLVSCQVASFTISSR	1.00	0.03
28	YFSLASDQGSVDGQLQLGIMYFSGNGVRKDYKMA[illegible]	1.00	0.03
29	ILKCVGEKMCVEVR	1.00	0.03
30	SYTHAKAGTLLCGFIKLLPMFLLVFPGMIAR	1.00	0.02
31	DCVTLTKAVMKARDACR	1.00	0.02
32	PELCEDR	1.00	0.02
33	PTRASSLSSVNSQNMK	1.00	0.02
34	LCDALDVFAK	1.00	0.01
35	NVLTNFYVK	1.00	0.01
36	ETERQTKRQTHTTSIHAHVCMFECTNVCK	1.00	0.01
37	REQNGQGDVLVNNAYSAVXAIFDNLGKK	1.00	0.01
38	CSDACACGKQCTEPK	1.00	0.01
39	PDKGQFPPDALGNEDPIWR	1.00	0.01
40	PDTSTPDSGEEMDPAR	1.00	0.01
41	RQSVFYASKGK	1.00	0.01
42	DALPWMHGDAIMSALK	1.00	0.01
43	MDFYEIHNMR	1.00	0.01

44	GGYSGPTCKVNVCK	1.00	0.01
45	DAEDATKALDGTR	1.00	0.01
46	EASLQLKMQTITKFECNSDSCNNR	1.00	0.01
47	CDMENAK	1.00	0.00
48	KALSGACDALMKK	1.00	0.00
49	LSVVSDASSNTTSGIVSDRMTVSLMMEMIIAER	1.00	0.00
50	TGTFTALDALYK	1.00	0.00
51	DRVNALIDLTDALK	1.00	0.00
52	SHFPDIVIPDGRPKKSK	1.00	0.00
53	MSTLSDMTCR	1.00	0.00
54	ACACGAENCSGFLGVR	1.00	0.00
55	NGLMMYTVNHEITNVASENSDNLK	1.00	0.00
56	CQVTDMK	1.00	0.00
57	SPLQNVYLPYVQRLVEVKLRIGHAMAR	1.00	0.00
58	ATVRPDAGQMRVFYGK	1.00	0.00
59	NYPNAYDVR	1.00	0.00
60	LMNSLKDTNSPK	1.00	0.00
61	TQCIDVDMCKGNPCTDGCVETEEGTGFLCTCPIGK	1.00	0.00
62	SSSVNLTKDNLKNMVNAPR	1.00	0.00
63	KDYQPYDRKDYQTPDR	1.00	0.00
64	HACLSGARCLMEMNKYK	1.00	0.00
65	VTCNLIACK	1.00	0.12
66	PFECEMCKK	1.00	0.32
67	DCVIACMK	1.00	0.30
68	EADMCCK	1.00	0.35
69	QYHCEICGNSFSR	1.00	0.24
70	IGCVSMFSSR	0.99	0.02
71	PTKAMCNLR	0.99	0.00
72	KCWSSLFTDR	0.99	0.03
73	WSTDLCR	0.99	0.00
74	KGICKSR	0.99	0.13
75	TSSQMCK	0.99	0.41
76	INTAAMCK	0.99	0.03
77	TRVLCMK	0.99	0.13
78	TNCHIMSEK	0.99	0.14
79	LNCHHKVCPR	0.99	0.01
80	IFCNFSICDK	0.99	0.03
81	MTCLRGK	0.99	0.00
82	LICELSQTHR	0.99	0.01
83	MCPSNTQAVSTMTR	0.99	0.00
84	ALMVNPDFFQELIFYKK	0.99	0.01
85	NCGAEHK	0.99	0.02
86	ERMSCAMKDYPESCAVLVRR	0.99	0.01
87	TQWSNCPTIK	0.99	0.00
88	MSMHTDGCGNR	0.99	0.06
89	VSTFNNCEDASK	0.99	0.00
90	LSSKDDCALR	0.99	0.02

91	MDCMNHK	0.99	0.06
92	NSLTEIHSCQR	0.99	0.01
93	EFCCNCGTVADR	0.99	0.07
94	MIPCSYK	0.99	0.05
95	SEANKCCAMR	0.99	0.07
96	VICINMLTFSR	0.99	0.05
97	VSICGGCNAMR	0.99	0.02
98	LASAVCSCR	0.99	0.01
99	EEICSCGK	0.99	0.01
100	YGNSCADNCSR	0.99	0.04
101	RQRYKGSLGASYK	0.99	0.00
102	EAYKSQMTPGSADVAKVTGQYK	0.99	0.00
103	KTRGAYK	0.99	0.00
104	RCQINCSIKSLDPLYKDMDAYK	0.99	0.00
105	MGSGGSKTPPSQQAVPDK	0.99	0.00
106	LSCRLLQCGDCK	0.99	0.01
107	SNNSSRSTMLSNSTVNSNMSSRNNSGRSR	0.99	0.03
108	NRVALSNMPVK	0.99	0.02
109	PLFRYTHLDKVECR	0.99	0.07
110	ECRYRVLAREVEAIG	0.99	0.02
111	SVIKKKYGQDACNVGDEGGFAPNIQDNK	0.99	0.05
112	SIGKMICNK	0.99	0.01
113	LEENGPVDLQTHEKGIEFNGTNAISK	0.99	0.01
114	RAQLRAAR	0.99	0.00
115	TLKDKADDPAMSIDYK	0.99	0.01
116	ELMKDYKNEVKDILAADK	0.99	0.00
117	TEKDYNIFNDLK	0.99	0.00
118	NIQKGDYNMVINDYEKAK	0.99	0.00
119	DYLATGSADY	0.99	0.00
120	NAYSADYMDYFQPK	0.99	0.00
121	IGIHCEWR	0.99	0.21
122	MPSRVVCFVCGNIGADLPLNIK	0.99	0.00
123	NHFHTVGPRTTNHVEGWHARMNK	0.99	0.01
124	SKKIYCLK	0.99	0.00
125	IVSSVSVR	0.99	0.40
126	GEGGGEGDEGDQR	0.99	0.05
127	MFNNGISFYGDFK	0.99	0.01
128	VSKSEPGVGDGSVSETMNSVTGSK	0.99	0.00
129	ASVSDPGVSEMK	0.99	0.00
130	RTLDRLRVQCR	0.99	0.00
131	MMNGSTHSRTNSWR	0.99	0.03
132	PIMQLSCSYK	0.99	0.00
133	LLPDNCSTANVSYK	0.99	0.01
134	PDSNDGNSDR	0.99	0.02
135	SMNCTLGK	0.99	0.02
136	ALGTELMCR	0.99	0.02
137	LKIPDSPKEMK	0.99	0.00

138	SPVCLAKVDK	0.99	0.01
139	GKGDTCTL	0.99	0.00
140	QCELSIGDGSVICRKK	0.99	0.01
141	GPESLSFITEYQKLLEKERNMDLNNLR	0.99	0.02
142	DPRMRQLCIK	0.99	0.00
143	LASDCTDKKMR	0.99	0.03
144	KTHMQSSLCEK	0.99	0.01
145	CPLRWYMLSSK	0.99	0.28
146	MLLILAMVLMSVTETDAVPLTK	0.99	0.05
147	VSQEQEHFLLNPFGTLYSEVSASSLVK	0.99	0.02
148	CGICGKAFSDGSNLIAHKR	0.99	0.01
149	LVLVKGKISSILLDMCEE	0.99	0.01
150	GANPDCR	0.99	0.00
151	LLADSNDCK	0.99	0.28
152	CISIALR	0.99	0.07
153	FTAGDCKK	0.99	0.05
154	SLFDSHNAFCRIAK	0.99	0.00
155	LTACRMGTEESR	0.98	0.00
156	CDIDHCISIK	0.98	0.13
157	CADMCSLSR	0.98	0.01
158	SSKHSSR	0.98	0.03
159	MTLGCGSRKGGFLSHSR	0.98	0.00
160	NYHLKMHSLTHSGLK	0.98	0.00
161	VSQVMKMYEDCLK	0.98	0.00
162	NFIMSGSTATCGCR	0.98	0.02
163	SASSCGK	0.98	0.02
164	MHTGENSLDCEICGK	0.98	0.01
165	PLETVLGR	0.98	0.19
166	IISLTPLGR	0.98	0.09
167	MSIPLGSLGFMR	0.98	0.09
168	LSKVYQVYKRGMALCGGELK	0.98	0.02
169	KVYKEIEEVVGTER	0.98	0.02
170	YSCSHNSK	0.98	0.01
171	RKAFCEK	0.98	0.07
172	MFCMTDDR	0.98	0.02
173	KTVEACIK	0.98	0.00
174	SQINKVARDLNLEANK	0.98	0.02
175	NEKNTIIVICMTNYSLIMNRLTK	0.98	0.01
176	DSREMRSDTCFTNR	0.98	0.01
177	LNMMNXKPLLPGVFYG	0.98	0.01
178	QPVEDPFIWIGQTFSTLYFMYFMLSPISSK	0.98	0.00
179	LAECVDSTPR	0.98	0.03
180	KGYRCHCGK	0.98	0.13
181	NGCLCCSVK	0.98	0.03
182	RAGNQCLLK	0.98	0.01
183	MDPLIARCCK	0.98	0.15
184	SDALDMR	0.98	0.04

185	DAALHMK	0.98	0.00
186	DAALHMK	0.98	0.00
187	EMALVSEQREADALEFKR	0.98	0.00
188	KCLLKICSPHR	0.98	0.01
189	QMIMKIHLAYLQHYEHIYEQSATNPVQCLSLIK	0.98	0.00
190	QCQPMDYSNDPNALR	0.98	0.01
191	IMGNRQHHCR	0.98	0.12
192	LTSALCQSFKRQMPDIGR	0.98	0.01
193	TWFQMCCQDISSAVR	0.98	0.13
194	CRHHDVHECKKMAEK	0.98	0.05
195	GIANCNAESSK	0.98	0.00
196	PAETNRRKTCNEVMK	0.98	0.00
197	TITYKVHSMESR	0.98	0.01
198	SVNHERMKQCLIK	0.98	0.00
199	METKTEGLCTR	0.98	0.00
200	MMKMSKVIGDKICYR	0.98	0.00
201	VRNLMLCK	0.98	0.07
202	PGNYCRR	0.98	0.02
203	MIYCHLGIGK	0.98	0.01
204	KARQSCRFDSSIGK	0.98	0.02
205	SMLCLDSNSVSEK	0.98	0.06
206	LCLAQISNTLLK	0.98	0.02
207	YTPCGLAWR	0.98	0.05
208	VCHPNVAKR	0.98	0.02
209	MLPNYCIK	0.98	0.01
210	LSCKSMGAAKYR	0.98	0.00
211	GAECKCPRGQALNGTK	0.98	0.01
212	RSHTIDNCLLK	0.98	0.01
213	SAQSLVDCK	0.98	0.22
214	HMPMNSGCRKGK	0.98	0.01
215	AFCLPTVSGK	0.98	0.00
216	PCFWLKHNMESDSYK	0.98	0.02
217	CMHNGQERQGIFRTECNICVCLK	0.98	0.02
218	QMMCSASDDR	0.98	0.00
219	LGISCAK	0.98	0.04
220	GHENNCPMLQSSTK	0.98	0.00
221	GCGSNGR	0.98	0.05
222	MFCADHK	0.98	0.05
223	SPAKSCEKTLTR	0.98	0.00
224	NRMSCSTVIQDRK	0.98	0.01
225	HYTDAYK	0.98	0.29
226	MESREYK	0.98	0.04
227	RISTGCMK	0.98	0.03
228	KFGASLVCCIPECGK	0.98	0.01
229	TMTSCFKSPR	0.98	0.04
230	AVMKCENTPDGK	0.98	0.02
231	SIECAHK	0.98	0.06

232	VPVQQMSTSSNSDWSRSR	0.98	0.02
233	PGSCQDRSR	0.98	0.02
234	LSGTPAFNDFCSALPSSHITAISFDGCQLADEDLVP	0.98	0.01
235	DFADINKYNWSLLKK	0.98	0.07
236	KKAIKYTALFTGK	0.98	0.05
237	RSNHDPSHEDYK	0.98	0.02
238	KTGAHNLCYKDPQMLK	0.98	0.01
239	MVMIWSIFICNK	0.97	0.01
240	FNLGMLFNFMK	0.97	0.03
241	NSLQPDCSDIR	0.97	0.08
242	QPDIYKNFK	0.97	0.02
243	AQAERAK	0.97	0.00
244	PQARTNSRDR	0.97	0.05
245	NITKIFCNK	0.97	0.01
246	SCDNSSTGNGNR	0.97	0.00
247	NGANVNNSTATNSTPLR	0.97	0.00
248	GHCGNPGNEAADR	0.97	0.08
249	AESSDYRR	0.97	0.04
250	ETDYRKKESMK	0.97	0.02
251	CRETNAEVALK	0.97	0.02
252	KNAAEGAKQTGCDRK	0.97	0.00
253	INSLVCWDWR	0.97	0.07
254	DPQARISTLCK	0.97	0.05
255	PMERCKISAQK	0.97	0.02
256	MKVILVCTSK	0.97	0.07
257	SLLTQDKC	0.97	0.05
258	MKFLLPLLCVGIVFAANHHGLTLDQISTMIIGNMD[illegible]	0.97	0.04
259	MCTIRMSFAK	0.97	0.01
260	DSNHTTPTK	0.97	0.00
261	GGGGEGR	0.97	0.34
262	IRPLMGGSGGGMQRPGPYDR	0.97	0.04
263	NHKSATAMSICK	0.97	0.02
264	IMIDFASRDCQVEFYEQMEK	0.97	0.00
265	LSDDFYQMKADFERRK	0.97	0.00
266	KQYSMNICHDGHTXQK	0.97	0.03
267	KSPSIPDK	0.97	0.02
268	RILIKNCTQK	0.97	0.01
269	PDTTMVEK	0.97	0.00
270	YMSPDSHKIHR	0.97	0.00
271	QPDDTVLK	0.97	0.00
272	EKCPYGFSCTDTWK	0.97	0.03
273	QCVQPPLK	0.97	0.00
274	NACSFSFDTVHFICDQETPLRTGLR	0.97	0.01
275	MFCYNGTAGQNRGCR	0.97	0.00
276	MSAIMDIK	0.97	0.00
277	MPQCQEYLSK	0.97	0.00
278	SSITSCKSLDK	0.97	0.03

279	RAAMSTACTLKGR	0.97	0.00
280	CASMIQR	0.97	0.03
281	QVSLSNGSLLVFMFLNFQRR	0.97	0.02
282	LVNSQRPASLLDLVNVGLTSIR	0.97	0.02
283	TFNCHVK	0.97	0.01
284	TPACGKPCGKITK	0.97	0.01
285	TKIIWGCK	0.97	0.04
286	LFCKSQVMIK	0.97	0.03
287	GQFSNVSCLGK	0.97	0.02
288	GSLHDSCLIIK	0.97	0.00
289	IFIDCKGK	0.97	0.03
290	VIHGAKLKSEDCK	0.97	0.01
291	REPYCVEKQEMIR	0.97	0.03
292	QRIVCPHQEGK	0.97	0.00
293	RITDELCQR	0.97	0.00
294	RCHRNADCQLDDNGDRK	0.97	0.00
295	QLEVLNMCMTR	0.97	0.00
296	SHTGEKPYRCEMCEK	0.97	0.00
297	MAMSTRCAIARSK	0.97	0.01
298	RCSDDTGATCSNR	0.97	0.00
299	SHTGEKPYLCSVCSK	0.97	0.00
300	IGLCNGTRLTMKKMMLK	0.97	0.00
301	PTCATKSQSSVR	0.97	0.01
302	LVIMNCPHPTVLSK	0.97	0.00
303	RVHYSCIK	0.97	0.00
304	KRYLGPLEKNK	0.97	0.26
305	ADVMPLGK	0.97	0.19
306	ILGPLIVDPSPDVR	0.97	0.14
307	PLGGSAK	0.97	0.11
308	LGAEEIAEKHNGERMGPLMR	0.97	0.10
309	FELSEPLGAK	0.97	0.09
310	PLGPSGGTASAIDDMSR	0.97	0.08
311	VCPHPLGELVAYCGTGEVVIMSDNGDPLYSR	0.97	0.07
312	LGRALKPLGSMPVTK	0.97	0.04
313	GEGCGMVILK	0.97	0.03
314	RKVYAMADARHEAK	0.97	0.03
315	IKIYKLDITMHPLGKTSK	0.97	0.02
316	TAVMKTDSRSR	0.97	0.02
317	QMFKMCVTKDIQR	0.97	0.00
318	LCPECQMVVNKELSK	0.97	0.02
319	VTGCIPN	0.97	0.05
320	LSSAVCQATDKK	0.97	0.02
321	MKPTCEGSFDR	0.97	0.01
322	SGGSCSK	0.97	0.00
323	TYNSLSGKGCPK	0.97	0.00
324	EIILDTLHFCMR	0.97	0.01
325	ATNGDCEK	0.97	0.00

326	TKTPEDCER	0.97	0.00
327	SNIFSSR	0.97	0.36
328	EKFEKQEYKDLTEFMADFR	0.97	0.07
329	RRHFSSR	0.97	0.04
330	YFSHEMRFK	0.97	0.04
331	MECGIESVWNNR	0.97	0.04
332	IYFSEASK	0.97	0.01
333	GKNIEHDLKNNFVR	0.97	0.00
334	AACSGEKMDR	0.97	0.00
335	PMTECIMKLILAK	0.97	0.03
336	ATCNDGLMTK	0.97	0.00
337	RIPKQLMCCELEGGR	0.97	0.08
338	IHTGEKPYVCEFCK	0.97	0.02
339	HDLILTNFETATYCNFCGKK	0.97	0.01
340	TINNAYTKVSIK	0.97	0.00
341	RINAAYSAK	0.97	0.00
342	NVSIAFPDDGACK	0.97	0.03
343	QKIEICSADQSCETK	0.97	0.00
344	KWNERGRMNQMCTAR	0.97	0.00
345	QCFEDNSVPDVRATWGR	0.97	0.00
346	KARFNHR	0.97	0.09
347	HLGELKAR	0.97	0.03
348	VNAICKRLIK	0.97	0.02
349	IKSASDKTRHCQSLK	0.97	0.01
350	FEALNLSFQNMCKXK	0.97	0.04
351	PATIDCYKNLRMVSLGDSNVR	0.97	0.01
352	LTYFVCRRKPDHR	0.97	0.00
353	ESELKKTDQAYK	0.97	0.01
354	LIESTYK	0.97	0.00
355	VMMRYKDSDK	0.97	0.00
356	AKDSISYK	0.97	0.00
357	LPATYKSK	0.97	0.00
358	NGHDALR	0.96	0.04
359	QDADALSLSMK	0.96	0.01
360	MKSTADALK	0.96	0.01
361	SAAYIDALR	0.96	0.00
362	PGAMCLNRLHNEAYER	0.96	0.01
363	GYVCLER	0.96	0.00
364	IYVCLDLTAQSSTK	0.96	0.00
365	AEVTEAMVCLLK	0.96	0.00
366	VTPGCDSVNK	0.96	0.01
367	SPMHESRCGGAVEKK	0.96	0.01
368	AIHLCISNQTGK	0.96	0.00
369	PIQNTFPEIMAQCM	0.96	0.01
370	QSCKTTLEDNLGRQP	0.96	0.01
371	DGKSLYCMSNR	0.96	0.01
372	DLTVGRVRRVMLK	0.96	0.00

373	NQHICAGELVDNGK	0.96	0.02
374	VGNGGCSQECVSDR	0.96	0.01
375	VVDCKCVYEK	0.96	0.10
376	DVNMCAEMLWIDTMR	0.96	0.02
377	INISTLIQLSSETQGCR	0.96	0.01
378	YLSDFLMCATK	0.96	0.01
379	YDVCGIGLIK	0.96	0.01
380	ITECDGGK	0.96	0.01
381	RNPNQMCPSMPRR	0.96	0.02
382	FHPLDACHFEVMR	0.96	0.02
383	GDTYLCVSATSDK	0.96	0.00
384	VSGTYVATTKYTKK	0.96	0.13
385	EGTKKLAQASKYK	0.96	0.07
386	EEGVILSKYKKQLK	0.96	0.05
387	MTDNKKK	0.96	0.03
388	CPMCTFVAK	0.96	0.02
389	IDXKKKDIEK	0.96	0.01
390	DSAPEMACLLGK	0.96	0.01
391	LDSPKKKMHNR	0.96	0.01
392	KKKASSIYGK	0.96	0.01
393	CRDLSSQTKKK	0.96	0.00
394	SLSMSNETKKK	0.96	0.00
395	WLPTESSEMSSTSER	0.96	0.00
396	CTPVMIAAWK	0.96	0.00
397	KELSFTDIETVSKMYGCADHCDR	0.96	0.03
398	TELTSCGQSLGTLR	0.96	0.02
399	RAECLEK	0.96	0.04
400	SCPITPRAK	0.96	0.00
401	TQDNMSRAQDK	0.96	0.00
402	MGCGASKSK	0.96	0.02
403	AGIKPYQCDFCGEK	0.96	0.00
404	PTCILLQEIK	0.96	0.00
405	TLMLDGEGYSCNR	0.96	0.05
406	QTSFNNNSCSSK	0.96	0.03
407	LVPSTFLAAMKKGMSLMGEGICSMK	0.96	0.01
408	SSIPMSCSKRR	0.96	0.01
409	DVEAETSCSPR	0.96	0.01
410	APQFLAPLMDYVYR	0.96	0.00
411	GCIKDIIHDSGR	0.96	0.00
412	GNGTCMSGR	0.96	0.00
413	YEVEQLEKKDAMSCPACSK	0.96	0.01
414	GSRSPACPR	0.96	0.05
415	MDCIQNAEGGK	0.96	0.01
416	QWMSWQPCTGR	0.96	0.02
417	NSNAMEPMGLIQCLSNMKK	0.96	0.01
418	MSHYRKMKER	0.96	0.00
419	LCCKRASLTGMAGK	0.96	0.02

420	PADCNSPSKK	0.96	0.02
421	ISAVLCTSGR	0.96	0.01
422	CRTSNLLIMMNNET	0.96	0.04
423	DYQITMDGNTHR	0.96	0.00
424	DITDYLMK	0.96	0.00
425	LHCEINVMETVNDVK	0.96	0.23
426	IGFTECELK	0.96	0.02
427	AGQRGDQECESAMK	0.96	0.02
428	SMLSKMKNQPSLCDIPK	0.96	0.01
429	DLCDGYMGK	0.96	0.00
430	LKKGKVATCQR	0.96	0.00
431	LAPKVLDGTRCK	0.96	0.01
432	NRAFTDIEKACQR	0.96	0.00
433	RCLLNDSNKDTVRK	0.96	0.01
434	GQCEFITDLKTTSLRK	0.96	0.00
435	PDLMGFHQCPQLR	0.96	0.00
436	THDRQFKFDICK	0.96	0.06
437	NEGGEGGK	0.96	0.03
438	MGGGRVGMGGMGMPMGRGGR	0.96	0.02
439	LACYTIEIYGTINK	0.96	0.00
440	MIDGKCVDFDECAMR	0.96	0.02
441	NDSPCGSTIGPIMSAK	0.96	0.02
442	SIPDIETDK	0.96	0.02
443	PDIRSTKKMK	0.96	0.02
444	IPDLDAK	0.96	0.02
445	SKKGHPD	0.96	0.01
446	NPDIMAGEK	0.96	0.01
447	KAPDGSK	0.96	0.01
448	PDRIIAR	0.96	0.01
449	DIDMSPDQMK	0.96	0.01
450	GKIPDGSLR	0.96	0.01
451	RVLRRMGYCTASDVIELK	0.96	0.01
452	GKGIPDLSR	0.96	0.00
453	FIPDGYLGITKEEAVTFPDGSR	0.96	0.00
454	WPDESKMCLNESQKR	0.96	0.00
455	KFNSYKCNSNHADLLSKK	0.96	0.01
456	QCQMATGQNDKK	0.96	0.02
457	RQNGGQTILCSRSPDK	0.96	0.00
458	GYCMIESGEGSSR	0.96	0.02
459	QVELCENGSGK	0.96	0.00
460	SFPCFDEPDMKAMFK	0.96	0.02
461	PRGILLLVDMR	0.96	0.06
462	LAGCLSVLNPNIALVLLRK	0.96	0.02
463	PRSGGNKKSCR	0.96	0.02
464	LRLTCRKAAGK	0.96	0.00
465	RCRHKAVYLNEAGR	0.96	0.03
466	PAGETVMIDIC	0.96	0.01

467	NVDSLMAFCHLHMQPR	0.96	0.01
468	FAEMCNIIER	0.96	0.00
469	KAILGCLKQAVQR	0.96	0.01
470	DICETSRMTITGDLK	0.96	0.00
471	GGRKILNSTTSMQACR	0.96	0.00
472	LKERLACLGDNIAR	0.96	0.01
473	LNCQRATNSQNIIQEKAMSLR	0.96	0.00
474	ACDGHGK	0.96	0.00
475	YKVLQQMVGCR	0.96	0.40
476	LAQECKSHFEKQSNK	0.96	0.08
477	LSMCSPFK	0.96	0.00
478	KAQHFAAECR	0.96	0.01
479	KGDDTVALVTDMSDQCR	0.96	0.00
480	NPPDQDPNAMTPSK	0.95	0.00
481	PNSPLGHSSIQGKR	0.95	0.14
482	TPLLGDQMERLR	0.95	0.10
483	TGKPPLGEVVIAYK	0.95	0.08
484	VKNSGFKLPLGTR	0.95	0.07
485	LLPLMLGAIDMSFPR	0.95	0.07
486	YFRGHHSPSR	0.95	0.07
487	LGGHAMPVTLPLMKK	0.95	0.06
488	ISIMSDIPTNKPLGILK	0.95	0.04
489	KVIDSNEMDKVMSILR	0.95	0.00
490	LTGNNWNLVACNK	0.95	0.00
491	SPQDLVLLMHCIER	0.95	0.11
492	NCSSELLMFRK	0.95	0.10
493	NMRYKNLAR	0.95	0.10
494	SNSELTSNVTYK	0.95	0.03
495	ILKTRYK	0.95	0.02
496	PWSHNYYK	0.95	0.01
497	VYMEYKNLAEHLTDPR	0.95	0.00
498	LIIEQYK	0.95	0.00
499	LHYKTDWHR	0.95	0.00
500	KAFLHWYT	0.95	0.46
501	TGESVSEKGCMGK	0.95	0.01
502	PWGIANDMTCSIR	0.95	0.01
503	FTTCSDDGTVR	0.95	0.00
504	LGGADNYKLCR	0.95	0.00
505	NMDEMAFCSKK	0.95	0.03
506	ALLKAACERHAK	0.95	0.00
507	CVLLDNK	0.95	0.12
508	ALEDAQKNYTRTAKELNR	0.95	0.08
509	SANAFSFVFSRK	0.95	0.06
510	PGNRVILK	0.95	0.00
511	SPGASALKICR	0.95	0.00
512	WASTFCQQTSYVIR	0.95	0.01
513	FCQSQERYLNMKMSK	0.95	0.01

514	VPLIMGILSCTK	0.95	0.01
515	FVISTCTQMK	0.95	0.03
516	LKYDPGLEGIDEDKK	0.95	0.07
517	VKTAKYTVLKK	0.95	0.05
518	SSEVAEDTSTSSR	0.95	0.02
519	KTLEGMKKK	0.95	0.01
520	FLRIDSPATKKK	0.95	0.00
521	IGCTGMHPVK	0.95	0.01
522	SMLLASSVIAHGNRMMGSRNCR	0.95	0.03
523	RIQLCELVDPERK	0.95	0.00
524	RVCDDAEAILNHR	0.95	0.00
525	SRLAGMDEDVHFTENCR	0.95	0.00
526	LKESCDSPIQCSETTANSTCNNETGVCQCIEDSISK	0.95	0.02
527	MSGNRCSPTEMRLGK	0.95	0.00
528	PSIKTVNCNGFK	0.95	0.00
529	VNNLLDEPGMDCRSS	0.95	0.00
530	LLKKSNYLMCPK	0.95	0.00
531	LASIKAR	0.95	0.03
532	FRCYSASLQGSELPNPK	0.95	0.02
533	NPTSDTNFSNSEAGK	0.95	0.00
534	SNSPAAETNR	0.95	0.00
535	AQIDVCSFYDR	0.95	0.01
536	DALQETNESDSLLMNK	0.95	0.01
537	ALDYIASK	0.95	0.00
538	LSMSSDSDYSEK	0.95	0.00
539	DYEKIGR	0.95	0.00
540	HTLSDDYEK	0.95	0.00
541	PSVITCAPMATER	0.95	0.01
542	AEIKQWYKGFMR	0.95	0.25
543	DPTGLTSAASRESR	0.95	0.00
544	CLMEQNVEER	0.95	0.00
545	CLAASEGAK	0.95	0.00
546	PTSASGHAALIYGCPFCGVR	0.95	0.01
547	TGCIQAGLRGK	0.95	0.01
548	RASDGGANIHCFQR	0.95	0.08
549	CCSLEHLRTDCKTSLKCSECNSTSHMTAMHMSK	0.95	0.00
550	GGEESSGGDGRLSRGNGR	0.95	0.02
551	NLTKPDFLR	0.95	0.01
552	LTFSPDVTSIK	0.95	0.00
553	WEMLKRAETACFR	0.94	0.04
554	LIPLCGNGIVER	0.94	0.02
555	SAREILCAMGR	0.94	0.01
556	PKVGDLCAAK	0.94	0.01
557	MSATGEGMKLAGFSALTFVAADDGTDSS	0.94	0.01
558	DARCVVESNGK	0.94	0.01
559	IRTRRCPQMAGK	0.94	0.00
560	QFEMIDGHLCER	0.94	0.00

561	VTWTKTLSFLLYK	0.94	0.03
562	CGNNPLIGR	0.94	0.01
563	LLVEPSMTHLAHIPDRSDRVTGNSLSLLER	0.94	0.01
564	ENTMNYKTIVTPKK	0.94	0.02
565	LYKTFKDRK	0.94	0.00
566	LSEKELIGTYK	0.94	0.00
567	NPGGTVLSYK	0.94	0.00
568	DPGAINITHMSSYK	0.94	0.00
569	SANSIMRIDKSYK	0.94	0.00
570	PEDATTASQVAEMYK	0.94	0.00
571	GGDIGAVYK	0.94	0.00
572	EICDLADKHK	0.94	0.02
573	KCVMGGEFEAALLK	0.94	0.01
574	MYIKHVQDMCIIWGR	0.94	0.00
575	PIFDTVCMLGVHR	0.94	0.01
576	CMESGGLSMMIR	0.94	0.00
577	TGSIHSGKDSR	0.94	0.00
578	VAMRTDANELTNPHSKSR	0.94	0.00
579	SLSPVMCSIVR	0.94	0.00
580	YENKDKMKSNVTIIVPCK	0.94	0.03
581	DTEILCKTGLYR	0.94	0.02
582	CSIEIGMK	0.94	0.00
583	MGANGLLCHVIK	0.94	0.00
584	LCHEVTLVAIKDGQER	0.94	0.00
585	GIVPITCGELFK	0.94	0.01
586	HVLTASHCFAEGTHAIR	0.94	0.13
587	PLEIPTAEESISTMLGK	0.94	0.07
588	APLATSTGSIDSLGNHLTFK	0.94	0.05
589	LPMLNPDLGASPLK	0.94	0.05
590	LGLLEPLSKEHK	0.94	0.04
591	KTSITPLQPMSSARCALGAVELDGK	0.94	0.02
592	ASEPLIKLNCMDK	0.94	0.00
593	EKLIFQIGTCDAK	0.94	0.00
594	MCQGFTTANNK	0.94	0.00
595	NSVASHEAGVSSATKKK	0.94	0.00
596	AGATKKPKK	0.94	0.00
597	GMSSTQKKNPLAMLKK	0.94	0.00
598	ISHDKYTMNWYHR	0.94	0.44
599	GSNGIIR	0.94	0.07
600	FNYEYDNLR	0.94	0.02
601	STVQESTGCSPNRIMLGR	0.94	0.00
602	DNYGGDLDALMKNK	0.94	0.03
603	LESTRHSKNFMSDKNESK	0.94	0.01
604	FSRAGGK	0.94	0.00
605	TEDCSDGLDEK	0.94	0.00
606	ANDAINMYTMCLEHIK	0.94	0.00
607	MDPSADITC	0.94	0.01

608	KIDPGYCDIFSPELAMPTR	0.94	0.01
609	EVKDTYMLQVIAKDGGSPMK	0.94	0.00
610	LVGLLDLKAGKDYVR	0.94	0.00
611	TGEMRVIK	0.94	0.00
612	GLCSPGMAFDTAR	0.94	0.00
613	NGSDVTYARAASNCR	0.94	0.00
614	NIQMFLTNSR	0.94	0.09
615	SNSGTNKTDGKAK	0.94	0.00
616	YLVSIISHK	0.94	0.00
617	VAVSHLQK	0.94	0.00
618	CRNMPQGSELRVPGK	0.94	0.00
619	DILSCLLEADPTKR	0.94	0.00
620	ATFAEKSAVCGR	0.94	0.01
621	CVVNGHTYCKMGLTGR	0.94	0.02
622	TKNQIDYIMINK	0.94	0.01
623	CHGGNVELK	0.94	0.01
624	LNDYEERKR	0.94	0.00
625	DDYEAIK	0.94	0.00
626	DYVAYIK	0.94	0.00
627	GQMSTADAEDYIK	0.94	0.00
628	CHDPASYMKIGK	0.94	0.00
629	GDYISKLK	0.94	0.00
630	HCDFSANTLGYMK	0.94	0.11
631	KMFDAKISEMEEICSK	0.94	0.00
632	CLPVNHSGHVTGR	0.94	0.00
633	VLSVRHVTMCDIMKR	0.93	0.00
634	LPASITAMEAIDYKPK	0.93	0.00
635	VKECLEK	0.93	0.05
636	SASDTCGMFGLSK	0.93	0.01
637	GGEHQGRGGREWDDRGR	0.93	0.07
638	KATLETVHAR	0.93	0.06
639	KAARHGK	0.93	0.01
640	SAALIQQCTAVKNK	0.93	0.00
641	LVDVGCIQHFTR	0.93	0.04
642	MPAYKLMNFDTKGR	0.93	0.01
643	SALIKAISSNYK	0.93	0.01
644	NGGISYK	0.93	0.00
645	FEEAYKALVSDKTK	0.93	0.00
646	EQMGGIMYKLSNMK	0.93	0.00
647	NTLVATYKGTK	0.93	0.00
648	IEAYKDSVEGK	0.93	0.00
649	AVDGSYVYK	0.93	0.00
650	EVTDEDVYK	0.93	0.00
651	NGDRVHVQWSDGAMYK	0.93	0.00
652	SMTGETNIPDEDK	0.93	0.01
653	SHGPDGK	0.93	0.00
654	SXELPDGQVITIGNER	0.93	0.00

655	SFSHSPDLTAHK	0.93	0.00
656	KPDESTLEAK	0.93	0.00
657	RSSPPDSNRYDHRR	0.93	0.00
658	VEITDALK	0.93	0.01
659	ANCNSGFENVPGNPSR	0.93	0.00
660	SAIADIMKEER	0.93	0.00
661	IPGVSVEECARR	0.93	0.01
662	DGNLVDCDPANK	0.93	0.00
663	LIVMGDGASGKTSLCMR	0.93	0.00
664	LGVASRPSCQR	0.93	0.02
665	TANCQPVGK	0.93	0.00
666	LAIEGKVIQRAECRPVANDTYMK	0.93	0.01
667	RHAIDWIPEKNILLLIVK	0.93	0.02
668	CAKSAFAMTK	0.93	0.02
669	DIIDCFVR	0.93	0.08
670	LASQNGCVTVPK	0.93	0.03
671	HYGQFTCEXCKSFFK	0.93	0.03
672	ENEVREILSPSECSSR	0.93	0.01
673	CIMRGANLPSEIDDK	0.93	0.01
674	LGGQECTGESVK	0.93	0.01
675	CIAGKHNGHK	0.93	0.00
676	LNAQLCVVDSGK	0.93	0.00
677	TVFCDGCALGVR	0.93	0.04
678	CMSTQKWQR	0.93	0.00
679	ELTIMTRCQERGTPLFK	0.93	0.00
680	TKPQVQINCWVTSNGKGR	0.93	0.00
681	CSLSGIK	0.93	0.00
682	CSFTIQMWNYHLR	0.93	0.00
683	AQTTRAADAVR	0.93	0.00
684	LEVKTGMDLGPLIYK	0.93	0.18
685	GSSNPLGLNSNLDK	0.93	0.13
686	XWLVPLMLGAPDMAFPR	0.93	0.12
687	TKFMNYSSTVVCTLGTSHPLMIETRDMFGNLTPYK	0.93	0.07
688	GMATALSMPLLGK	0.93	0.02
689	VYECMFKVLKDVELLDDR	0.93	0.02
690	LTLLSAQSHGCAGIEK	0.93	0.02
691	YNLPINTMWIQRDSCEDNKRFK	0.93	0.02
692	KEDATCPVSEPGSTLGKIK	0.93	0.00
693	SFVSFLGK	0.93	0.01
694	ILTANEMACALFGYNR	0.93	0.04
695	NITLSGPTTFCPIIEK	0.93	0.00
696	KDYNLPKPKMTNSDLSR	0.93	0.00
697	YMSVCLLYRGDVISK	0.93	0.00
698	RFFSGCTIPR	0.93	0.18
699	MSTNSSDLYVR	0.93	0.01
700	EESSPAKISFLDAQR	0.93	0.01
701	IVSIATNSNNRQVERIMK	0.93	0.00

702	KGIAMTVSHQQSMPK	0.93	0.00
703	ICYVGIKTVK	0.92	0.00
704	SFSRRGALAIHK	0.92	0.01
705	QSNAENKNTKNSNTEGK	0.92	0.00
706	ALKLYVYNTDSDSCR	0.92	0.00
707	DYTEDIQIDASSRYR	0.92	0.02
708	PKFIDKFLLGASSLSRK	0.92	0.00
709	KRGKESDYMIGR	0.92	0.00
710	SGDYEEDMER	0.92	0.00
711	FTSCQAPLVTADDR	0.92	0.00
712	LTAISLGQGQGPRAEAMMR	0.92	0.02
713	FFYIPFGQGPRICLGMRLALLELK	0.92	0.01
714	TGKMHRFAFCVK	0.92	0.00
715	ICETSREVVAIGK	0.92	0.01
716	RCFYRPLTPR	0.92	0.00
717	AVRGCQGVDLNR	0.92	0.05
718	LCSVANKIQACYGFFSWKK	0.92	0.01
719	FHFDDKLSYK	0.92	0.02
720	DRDEEREYKSSR	0.92	0.01
721	GIQHKGYK	0.92	0.00
722	ASLRGDVDAYKMEK	0.92	0.00
723	YKDIGKASMKNLPISSSK	0.92	0.00
724	GPQLICSGSDDGTIK	0.92	0.00
725	CSCFDTIFKGTYENGGIVR	0.92	0.01
726	IKAGRELGLTFVPFPEGGGR	0.92	0.02
727	YGPVCFVDMKSR	0.92	0.01
728	LPDVRASSTRK	0.92	0.03
729	PRSPDPVEGR	0.92	0.02
730	LTYIPDELGR	0.92	0.01
731	TAYEMAQSMDKRVSYICLINS	0.92	0.01
732	VIEFPNPDK	0.92	0.01
733	GFHGDGYNSCTK	0.92	0.00
734	MMYLSVPDMMGK	0.92	0.00
735	EDPPDTSGIKMDR	0.92	0.00
736	LAGPDLK	0.92	0.00
737	HLANGLPDFLMK	0.92	0.00
738	TSPSSFGGDDICQK	0.92	0.01
739	DGLDIMLNSLCEMAGK	0.92	0.00
740	LAILIRSR	0.92	0.35
741	ERSHSFSSPSLQK	0.92	0.02
742	EMKMKKK	0.92	0.10
743	DALGAPK	0.92	0.01
744	DEDALRGLDPR	0.92	0.01
745	CPSGSQSSKSHMAR	0.92	0.01
746	TLFPLYTDAQCDGLINDYESAVNKKGK	0.92	0.00
747	KENSGVEGCLLGK	0.92	0.01
748	LMLKYCDIFGILDK	0.92	0.01

749	VLVNRGGKSIQDECEQYITK	0.92	0.00
750	QVTSKLEQALQHYDDA	0.92	0.00
751	LNKGGMFGSYTPDRYK	0.92	0.00
752	LVGMSTGNVSLKEHVHPDLAGCADK	0.92	0.02
753	MSCNFPHGGAMDTHFANMR	0.91	0.00
754	AADDVGSCGMDRAK	0.91	0.01
755	VKNQRMCFRTNGTK	0.91	0.04
756	GGVPSMLRLASCPEKSR	0.91	0.00
757	SLIRNSKTNK	0.91	0.02
758	SSHYLLVMKG	0.91	0.01
759	ILSLQAAHIVSHMIK	0.91	0.00
760	PLGKKNEGFCSGK	0.91	0.07
761	NPLGLVPVLEKDGK	0.91	0.05
762	VQELGKNLGMEINR	0.91	0.01
763	SPHATIDYLNK	0.91	0.00
764	CRIGGASSFR	0.91	0.00
765	ARGESSSDYAWK	0.91	0.00
766	TTDTIEVSKLNGDYRK	0.91	0.00
767	PVDYPAGR	0.91	0.00
768	IVSTYKSRNDEFRK	0.91	0.08
769	IDSPLILYK	0.91	0.01
770	VVLRSYK	0.91	0.01
771	VERAHRQISGAYK	0.91	0.00
772	EPAQAYFKDQIYK	0.91	0.00
773	VSGCEGARWR	0.91	0.02
774	CLEMEKSIKASSDNEVTLR	0.91	0.00
775	FKFSLSTSSR	0.91	0.00
776	QLFPPTFSSLSR	0.91	0.00
777	GDSVWKCVGLVK	0.91	0.03
778	GWQPVNICITSAGDLLVTMR	0.91	0.01
779	FHTCVSLLEGMVNPER	0.91	0.02
780	NEGLKMMMSLIK	0.91	0.00
781	GIPDPYLRER	0.91	0.01
782	SIEQNMSMIGKMVIDMKDPDR	0.91	0.00
783	TLISAPDDRPTSK	0.91	0.00
784	PDLESGEASLNTTPR	0.91	0.00
785	SADSKEPDSKTTK	0.91	0.00
786	GKRPSCATLFAANK	0.91	0.00
787	FAHCGVVAMR	0.91	0.14
788	ALERAINKPSYTCTR	0.91	0.01
789	SAIALEK	0.91	0.02
790	NMVDVSAIASIMPR	0.91	0.00
791	LADITEPNAIGK	0.91	0.00
792	YVLRNGGIRR	0.91	0.07
793	ANMLGCISLSAR	0.91	0.02
794	KSCSMKLVVK	0.91	0.02
795	GGISLPVFRCAR	0.91	0.03

796	CGQKVAQATTLLK	0.91	0.01
797	RCMGNAHQTLKVLSDLLGHDNTEIR	0.91	0.00
798	LGCHSGFQTWVK	0.90	0.03
799	ERGKDKDSSSK	0.90	0.00
800	LLDDSTNENGSLKAR	0.90	0.03
801	LSPKAARKDR	0.90	0.01
802	CEAEMAGVDIK	0.90	0.00
803	IAYVLTWANSCQNSTVCSYYTPYKGHPGEKDFLK	0.90	0.01
804	MGTKQSHIHKLDTVSKR	0.90	0.00
805	SLAALDATNR	0.90	0.01
806	NDSKMAVDAALR	0.90	0.00
807	EVVDMVCLNADK	0.90	0.01
808	SKIDYHKVNMEPLDK	0.90	0.00
809	PKEWTDHDYALQNSNESSK	0.90	0.00
810	KIDWIKGIDYRR	0.90	0.00
811	KFLEYKFGQGVVENPR	0.90	0.00
812	QYVEGLMSDYQK	0.90	0.00
813	HSMGHRNMTDYSHKSSK	0.90	0.00
814	NMDEPQRGDYVHAKIK	0.90	0.00
815	GDDYILNGQK	0.90	0.00
816	GRPIGSSFR	0.90	0.03
817	VLHHQSVACQRKPMSPLELATMAR	0.90	0.01
818	SPLGSPR	0.90	0.32
819	RHNRVGMPLGSMPIPK	0.90	0.12
820	FFLGGPLTLRGFNIK	0.90	0.05
821	VLYTYKASEDMAATESMPLLGYEVTRLSTWFEGCK	0.90	0.02
822	MTTVGVNASQPDRVDALR	0.90	0.01
823	GTPTLTQFFNKKDCK	0.90	0.02
824	MKSDNKGVGCSK	0.90	0.01
825	LSAASLLDCPNARKMSSVSLMESPYRR	0.90	0.01
826	EISILEGEMYQLSHMLSDQKILMSSMMAMSLVSDK	0.90	0.00
827	KMAGFSR	0.90	0.02
828	MLYETGIFSR	0.90	0.00
829	FQHRCVSVFMDKK	0.90	0.02
830	SPDGLITMNNFR	0.90	0.11
831	LGFEPDRK	0.90	0.05
832	TLGAEKRSRPDDTESTIVMR	0.90	0.01
833	KLVARVSEQMANPDMGR	0.90	0.00
834	NAMPDVLDPVK	0.90	0.00
835	TKASKVTSFPDAGK	0.90	0.00
836	VNGALPDTTSNVTDSNK	0.90	0.00
837	ISEGNLMSGTVPDIR	0.90	0.00
838	APHATSNNRYVVCLEK	0.90	0.00
839	IRKLFNLXKEDDVR	0.90	0.03
840	MFNLLEIDSTK	0.90	0.01
841	NSAIMSDVK	0.89	0.00
842	IEMAAIKSAELSSIMSKK	0.89	0.00

843	QNCIVFLSNK	0.89	0.01
844	ICGQSFFQRSLFTSHK	0.89	0.05
845	YKVDGISRDFR	0.89	0.21
846	NYKFKIRFR	0.89	0.08
847	VLGRKSGYKLR	0.89	0.00
848	EAIFYYSYK	0.89	0.00
849	CISEGAPSQSKENFLK	0.89	0.00
850	TNSWTQVANMSGR	0.89	0.00
851	ASVGHSR	0.89	0.07
852	YTLSQETVDYVK	0.89	0.00
853	ELNINDRACVKLSYLPK	0.89	0.01
854	LANLLGDTIFPKPIDLKVPLFK	0.89	0.03
855	KQAASRFYTLLVLK	0.89	0.01
856	RYWPVIDDALRR	0.89	0.01
857	LYTVQFQDALR	0.89	0.01
858	MRFVIDKMSDVSESESCSIHK	0.89	0.00
859	IRVSSLLMFMMAAR	0.89	0.03
860	MEPDGIMNIFRRSEK	0.88	0.07
861	VVNLPDAWTK	0.88	0.02
862	ISLQLPDIKMNNLLTDK	0.88	0.01
863	DITAQIKRLNIPDK	0.88	0.00
864	KFAWPDK	0.88	0.00
865	EPDVILTQTIKGK	0.88	0.00
866	QQSFIMAVSSKPDKIK	0.88	0.00
867	MLGKLYFSK	0.88	0.00
868	LGAARSKTQMFSR	0.88	0.00
869	VVNMYKSVR	0.88	0.03
870	EAYKRADEVLQSHK	0.88	0.01
871	VKTAEVATYRYK	0.88	0.00
872	GVDQIMAEIYK	0.88	0.00
873	MEGKSYKGVKK	0.88	0.00
874	MSKEEEKDGMAKDGNK	0.88	0.00
875	TCKSLWLVIDNK	0.88	0.02
876	CASTMATTYSSGSIDDK	0.88	0.01
877	CGSATPGASSQNGK	0.88	0.01
878	KEAKAQAATNSGEDAFEMGIK	0.88	0.00
879	CIVKILSDKANLMK	0.88	0.00
880	LPIYMMLVDYQKIVR	0.88	0.02
881	DGDYFADMEGR	0.88	0.01
882	AISGGEITDYDVMESEK	0.88	0.00
883	KFSISSQDKFVTVMK	0.88	0.00
884	APLVMTFCVFEGSK	0.88	0.03
885	YMSCIMLYRGDVVPK	0.88	0.00
886	NLKLGSELGK	0.88	0.00
887	VSLLYDVPGYK	0.87	0.02
888	MSALQLKDGFYK	0.87	0.00
889	GLVTETSVLDPDEGIR	0.87	0.01

890	PDPSELNTQSKKQKGENNSDSGSDK	0.87	0.00
891	AGSTPDLQEKDQR	0.87	0.00
892	VQIAPDSGGMPER	0.87	0.00
893	GYYTGDPDKRR	0.87	0.00
894	LKFSISRSHLEQMSFIGK	0.87	0.00
895	SFEGAKSIKYFSDR	0.87	0.00
896	DDMGLCELFSPSKVFD	0.87	0.03
897	SVSFVDESHK	0.87	0.00
898	ARMQEAADNLGSTSSDYSFTISK	0.87	0.03
899	VKIPVGNSMMVDYR	0.87	0.00
900	VFTQDYDNNSDR	0.87	0.00
901	VDYIKAHMTSQFGTVLK	0.87	0.00
902	IDYNFGDLER	0.87	0.00
903	CITEHFRNVYSSSK	0.87	0.04
904	FMTLDQIVVDEKFSECRR	0.86	0.02
905	IPPKPFPLGYAKEK	0.86	0.09
906	LFRSLAMTTPDR	0.86	0.02
907	SYEXPDGQVITIGNER	0.86	0.00
908	IEPDVGMSQLDANR	0.86	0.00
909	KYELPDGQVITIGNER	0.86	0.00
910	RHAARPDLMVK	0.86	0.00
911	MAEKSHNYNVPDIIEAGQK	0.86	0.00
912	MLFTGSFCLTQKEMTDLVVK	0.86	0.01
913	PGVLAIIANNAAIGIVTSLFLR	0.86	0.00
914	NTSPSALDATGSSTKNPK	0.86	0.00
915	LFSVLLSRTK	0.86	0.00
916	MGINQTAHFSMETPVAISR	0.86	0.00
917	FYKQHASHGFLTKLR	0.86	0.01
918	EVSVMAKAHSDVDISR	0.86	0.00
919	TVLFVPEPHVCERLIR	0.86	0.00
920	VNAQQGSPENNAAFR	0.85	0.04
921	IDVKLNPDR	0.85	0.00
922	AIRLDPDSKLMQQEMQNLTR	0.85	0.00
923	TNSYPNIVFDHYR	0.85	0.00
924	TATTADYNNLGAEIGR	0.85	0.00
925	WGVMSGDITPQDYNRR	0.85	0.00
926	KALGKKTMSEGDLAR	0.85	0.01
927	ILQVLLENDGTFGRYK	0.85	0.02
928	VLHPVRSMGVGSTYK	0.85	0.00
929	QGSPVGNKMPTAKPNYK	0.85	0.00
930	FRFNLFMYSALYRK	0.85	0.15
931	CIYNLVDKPLPGEK	0.85	0.01
932	MYTQMTDESQDALLPR	0.85	0.00
933	VVPDILIEQGK	0.84	0.01
934	PANIMFADNNCPVLMDLGSSVMARTEIK	0.84	0.00
935	CHTFIGGMPVVEDK	0.84	0.00
936	LNLAGRDLTDYLMK	0.84	0.00

937	QLLMAMVVNKTTIADNYK	0.84	0.00
938	LDNSNSIMSEIR	0.83	0.01
939	LQWDPDHSPAGGKLVR	0.83	0.01
940	YYGFMFDTTPDRAHR	0.83	0.00
941	IKGTDTLILLDYLK	0.83	0.00
942	MMDKHGEDYVAMARDPMNIFQHTPK	0.83	0.00
943	RAQRFVVK	0.83	0.00
944	DQNHTHQMVLQKAFVEKGQR	0.83	0.00
945	FPDDVVMIEKVR	0.82	0.01
946	IEKYVPDQEVQQSQK	0.82	0.00
947	YGCKWSTIEGFQFVIKEQVK	0.82	0.01
948	LYVVQVENALLNR	0.81	0.21
949	PDGVFISR	0.81	0.00
950	HMPDVAEGVGK	0.81	0.00
951	PDGVFIINLR	0.81	0.00
952	RLCGVFFPSLMETGR	0.81	0.01
953	FFSTLYASMSR	0.80	0.00
954	RLRVFSRMESEQGAEK	0.80	0.00
955	PDTVTENTFLDWPVVMNGK	0.80	0.00
956	PDTRIVNNSFFINERAR	0.80	0.00
957	GFAFVTFEDYDSVDK	0.80	0.00
958	SLLVGQYEANPLIHDEMQK	0.80	0.03
959	CAPEFTVFMASER	0.80	0.02
960	IKAYFGKEGSSR	0.79	0.00
961	TLFFSRLDRMVEREWR	0.79	0.00
962	TFLLVFGKK	0.79	0.03
963	LXSLPVLAGAITMLLTDR	0.79	0.01
964	DARTEDKDIFGDTALLAATR	0.79	0.00
965	SIEVGDDYNIPHWMNHSFYMSK	0.78	0.01
966	LFVTQVEDALVGR	0.77	0.01
967	GYDVFKAYARSK	0.77	0.04
968	MTSVFGGSKPDSQLLTK	0.77	0.00
969	IDIGVGDALTSGGA	0.76	0.00
970	XSRVAELANAVVSNADQK	0.75	0.01
971	IMVFAVQHGK	0.74	0.00
972	VVFINKTNILLEQQYKR	0.73	0.01
973	CATSVQRRWRGFLGR	0.72	0.00
974	AYVNFSK	0.68	0.02
975	FTVALSK	0.67	0.01
976	AMSKDVNR	0.67	0.00
977	PKIPSKK	0.61	0.01
978	QEEIMSAKNR	0.59	0.01
979	ENVNVVHEAFR	0.00	0.29
980	SRMDSRK	0.00	0.18
981	GGGGGRPR	0.00	0.17
982	MSCEMSSSPR	0.00	0.17
983	RLDPVTQDPERMCCK	0.00	0.10

984	SSSCYTLQELR	0.00	0.09
985	IMDIIRAQEQSR	0.00	0.08
986	SQMMTDKCQDK	0.00	0.07
987	RNAFSAIKFGLNR	0.00	0.07
988	CPSSGSLLR	0.00	0.07
989	LNQMVFLGYTNAVK	0.00	0.06
990	LGCLIMLIR	0.00	0.06
991	PVVGRISGGGER	0.00	0.05
992	IEQSSPTHNGCPEKRPK	0.00	0.04
993	YLMHMDKK	0.00	0.04
994	SMFLDLTDCQTIMTCK	0.00	0.04
995	TIAECLADELINASR	0.00	0.04
996	IRPCFEKDKSQVR	0.00	0.04
997	AVFKDSFCR	0.00	0.04
998	MSASKIFTGYRALGYVSNHVPLVTR	0.00	0.03
999	GRGGGAIHGDR	0.00	0.03
1000	TRVSLQYMCQLISEIPR	0.00	0.03
1001	GGRGGMGGGR	0.00	0.03
1002	TLLRIFLHNHHGNPNK	0.00	0.03
1003	PSNATIVFKDPR	0.00	0.03
1004	VLLSTLRGALEEAKNR	0.00	0.02
1005	SFVMSAIIAEGVKPTLAELEK	0.00	0.02
1006	LGQSAVKNICMK	0.00	0.02
1007	RLLLFGILLLMGSVSSRR	0.00	0.02
1008	AGTMSKK	0.00	0.02
1009	SEICDITSNMLATIVNVDVKDSLTSEFRKMMPLCK	0.00	0.02
1010	DTVKPVMCAYK	0.00	0.02
1011	RLSESDKK	0.00	0.02
1012	CNVKKTV	0.00	0.02
1013	LSDFGFCAQVTPELR	0.00	0.02
1014	MAYTADSNAPLRDVKK	0.00	0.02
1015	RTIANYVMSR	0.00	0.02
1016	HVNKPNVSKSESLSSSKLNR	0.00	0.02
1017	LILESFHGLIPDNVKSLIMK	0.00	0.02
1018	KAAMARGSLYSFVPDR	0.00	0.02
1019	VTDKPNSNEMKK	0.00	0.02
1020	LVRGVSDCIGSNYSPR	0.00	0.01
1021	VSPDATVEALLR	0.00	0.01
1022	NPDMSMSEAKTLIMK	0.00	0.01
1023	NYETKQSKK	0.00	0.01
1024	TKCMDIIEVVSIWEKREAMK	0.00	0.01
1025	EKMEVKAAQLAGEMASMKAR	0.00	0.01
1026	SQRVADMVDK	0.00	0.01
1027	CEARRVIFGK	0.00	0.01
1028	GWFPQALIDANMAKVLMDFLNDLR	0.00	0.01
1029	DISGVSPKCTDSK	0.00	0.01
1030	GTVRYKGPVATKK	0.00	0.01

1031	KPSSPLR	0.00	0.01
1032	AKDKDTMSDIGGGSSKK	0.00	0.01
1033	MMGNKLCAPLLSKK	0.00	0.01
1034	LDALKTMLAYPDVR	0.00	0.01
1035	VKICNKPK	0.00	0.01
1036	GYQAIAAYHGVPAKCR	0.00	0.01
1037	IACSVSFLHGAMFHFIVVCMMDR	0.00	0.01
1038	DSKKMYAMK	0.00	0.01
1039	SFSCRSNLAHHK	0.00	0.01
1040	VIVVAILVTGKAQCVCLTVP	0.00	0.01
1041	MCFCTFSSVLRLQQR	0.00	0.00
1042	ETKNTISHR	0.00	0.00
1043	KGGLASKCVDDQELQQLR	0.00	0.00
1044	ESFSPRSITTDSQSR	0.00	0.00
1045	XXKSMPLK	0.00	0.00
1046	VPKYDNPTGISHYLILK	0.00	0.00
1047	ATERECATMNDMPYLSLK	0.00	0.00
1048	NPDMSMSEAK	0.00	0.00
1049	SKSVDDFLDSDIDTNANCNSK	0.00	0.00
1050	YDIKVDHR	0.00	0.00
1051	HSKSTATKVDR	0.00	0.00
1052	EDILAYNQWLEGFSFSGQQKK	0.00	0.00
1053	DSGIVMTPPPDSTTTPDVQR	0.00	0.00
1054	LSILYTNIPSGGSRSHAK	0.00	0.00
1055	KASPSKTSVEKSIVSSVPK	0.00	0.00
1056	CRSITSYFSQAEGK	0.00	0.00
1057	SMTGETNIPDEDKMRLLR	0.00	0.00
1058	YMDFNENGSCVIGGNKCLSDKYIR	0.00	0.00
1059	SLDYGSTSAVVPQK	0.00	0.00
1060	AETKGKKSPR	0.00	0.00
1061	MVQPTMDILQCDPK	0.00	0.00
1062	VSTQWMPQPLTPDQK	0.00	0.00
1063	INSMAKVFYLFLMILQMLK	0.00	0.00
1064	MVDIGKTASSKKK	0.00	0.00
1065	MTSKPTNSRVIK	0.00	0.00
1066	PSPGNYDR	0.00	0.00
1067	SQHTNQAVFSINHYAGK	0.00	0.00
1068	GRSVNLKSASYKVIR	0.00	0.00
1069	SLQQGQWMGVDYR	0.00	0.00
1070	KDDMEIVCERFTTSK	0.00	0.00
1071	EISTGANSSKRRPNAXNK	0.00	0.00
1072	MVLEDDGRFNFMLQPIR	0.00	0.00
1073	YEAKLQPDSEFSPQFK	0.00	0.00
1074	GGSGVPDFASIK	0.00	0.00
1075	TLKLETVTPDR	0.00	0.00
1076	NQQEKVAEMEQRIR	0.00	0.00
1077	LCEDFERDMK	0.00	0.00

1078	MPLSMSHK	0.00	0.00
1079	HTGKFMDGDDPDPKR	0.00	0.00
